# Comparison of Neurodegeneration and Cognitive Impairment in Neonatal Mice Exposed to Propofol or Isoflurane

**DOI:** 10.1371/journal.pone.0099171

**Published:** 2014-06-16

**Authors:** Bin Yang, Ge Liang, Soorena Khojasteh, Zhen Wu, Wenqiong Yang, Donald Joseph, Huafeng Wei

**Affiliations:** 1 Department of Anesthesiology and Critical Care, Perelman School of Medicine at the University of Pennsylvania, Philadelphia, Pennsylvania, United States of America; 2 Department of Anesthesiology, Shanghai First People's Hospital, Shanghai JiaoTong University School of Medicine, Shanghai, China; 3 Department of Anesthesiology, Tongji Hospital, Tongji Medical College, Huazhong University of Science and Technology, Wuhan, China; 4 Department of Neurology, Dongfeng General Hospital, Hubei University of Medicine, Shiyan, Hubei, China; Imperial College London, Chelsea & Westminster Hospital, United Kingdom

## Abstract

**Background:**

While previous studies have demonstrated neuronal apoptosis and associated cognitive impairment after isoflurane or propofol exposure in neonatal rodents, the effects of these two anesthetics have not been directly compared. Here, we compare and contrast the effectiveness of isoflurane and propofol to cause neurodegeneration in the developing brain and associated cognitive dysfunction.

**Methods:**

Seven-day-old mice were used. Mice in the isoflurane treatment group received 6 h of 1.5% isoflurane, while mice in propofol treatment group received one peritoneal injection (150 mg/kg), which produced persistent anesthesia with loss of righting for at least 6 h. Mice in control groups received carrying gas or a peritoneal injection of vehicle (intralipid). At 6 h after anesthetic treatment, a subset of each group was sacrificed and examined for evidence of neurodegeneration, using plasma levels of S100β, and apoptosis using caspase-3 immunohistochemistry in the cerebral cortex and hippocampus and Western blot assays of the cortex. In addition, biomarkers for inflammation (interleukin-1, interleukin-6, and tumor necrosis factor alpha) were examined with Western blot analyses of the cortex. In another subset of mice, learning and memory were assessed 32 days after the anesthetic exposures using the Morris water maze.

**Results:**

Isoflurane significantly increased plasma S100β levels compared to controls and propofol. Both isoflurane and propofol significantly increased caspase-3 levels in the cortex and hippocampus, though isoflurane was significantly more potent than propofol. However, there were no significant differences in the inflammatory biomarkers in the cortex or in subsequent learning and memory between the experimental groups.

**Conclusion:**

Both isoflurane and propofol caused significant apoptosis in the mouse developing brain, with isoflurane being more potent. Isoflurane significantly increased levels of the plasma neurodegenerative biomarker, S100β. However, these neurodegenerative effects of isoflurane and propofol in the developing brain were not associated with effects on inflammation or with cognitive dysfunction in later life.

## Introduction

Studies using a variety of animals ranging from rodents to rhesus monkeys have shown increased neuroapoptosis during postnatal brain development after exposure to intravenous or inhaled anesthetic agents [1]–[9]. General anesthetic (GA)-mediated apoptosis in the developing brain is correlated with an elevation of plasma S100β, a neurodegenerative biomarker in blood [10], [11]. Furthermore, some studies suggested that the anesthetic-mediated apoptosis in the developing brain may be associated with persistent learning deficits and social behavior dysfunction [1], [2], [12], although such an association could not be confirmed by other studies [10], [13]. Most importantly, multiple exposures to general anesthetics in children under the age of 4 may be related to learning disabilities, including reading, language and math [14]. The mechanisms of general anesthetic-mediated apoptosis in the developing brain are still not clear, although many hypotheses have been proposed, including the disruption of intracellular calcium homeostasis [13], [15]–[26], activation of gamma-aminobutyric acid receptors and inhibition of N-methyl-D-aspartate receptors and associated impairment of synpatogenesis [1], [25]–[29], activation of P75 neurotrophin receptors [5], [30], regulation of cell cycle [31], and others [32], [33].There is increasing evidence suggesting that GAs may cause apoptosis and cognitive dysfunction by aggravating neuroinflammation [12], [34]–[37], although surgery itself may cause cognitive dysfunction via increased inflammation [38]–[41].

General anesthetics, however, may not all affect neuroapoptosis in the developing brain or subsequent cognitive function with the same potency. This has important clinical implications for our pediatric patients [42], [43]. Among the commonly used inhalational general anesthetics, isoflurane is the most widely reported to induce neurodegeneration in the developing brain and subsequent cognitive dysfunction in several animal models [1], [2], [13], [28], [30], [44], [45]. Similarly, the intravenous general anesthetic, propofol, at clinically relevant concentrations and durations, has also been shown to cause significant apoptosis in the developing brain [4], [5], [46], [47], and associated cognitive dysfunction [7]. While comparisons of different inhaled anesthetics to induce neuroapoptosis in the developing brain and subsequent cognitive dysfunction have been undertaken [10], [12], [48], [49], such a comparison has not been carried out between an inhalational anesthetic and propofol. Because propofol is the most commonly used induction agent for general anesthesia and isoflurane is the most widely studied inhalational general anesthetic, this study compares the potency of propofol and isoflurane to cause neurodegeneration in the developing rodent brain and associated changes in cognitive function.

## Materials and Methods

### Animals

The Institutional Animal Care and Use Committee at the University of Pennsylvania approved all experimental procedures and protocols used in this study. All efforts were made to minimize the number of animals used and their suffering. All C57BL/6 mice (Charles River Laboratories, Wilmington, MA) were housed in a University of Pennsylvania animal facility in polypropylene cages and the room temperature was maintained at 22°C, with a 12-hour light-dark cycle. Mice had continuous access to water and food. Both male and female mice were used in the experimental and control aspects of this study.

### Anesthesia exposure

For the isoflurane (2-chloro-2-(difluoromethoxy)-1,1,1-trifluoro-ethane) exposure studies, postnatal day 7 (P7) mice (n = 22) were placed in plexiglass chambers, resting in a water bath to maintain a constant environmental temperature of 37±0.5°C, and exposed to 1.5% isoflurane in humidified 30% oxygen/70% nitrogen for 6 h. Five liters of total gas flow were used to ensure a steady state of anesthetic gas and prevent accumulation of expired carbon dioxide within the chamber. Isoflurane, oxygen and carbon dioxide were monitored and maintained using IR absorbance (Ohmeda 5330, Detex-Ohmeda, Louisville, CO) as described in our previous study. Mice in the control group (n = 18) received humidified 30% oxygen balanced with nitrogen only for 6 h at room temperature. All animals were monitored and stimulated every 30 minutes to ensure reactivity. They were assessed constantly by observation to ensure the adequate spontaneous breathing same as in our previous study [10]. We did not monitor blood gases in these treatments as previous study has demonstrated that 1.5% isoflrune for 2 hr did not affect arterial blood gas significantly [50]. 12 male and 10 female mice were used for isoflurane treatment group, while 10 male and 8 female were used for corresponding controls.

For propofol (2,6 diisopropylphenol) anesthesia, mice (n = 20) received one peritoneal injection of propofol in intralipid (150 mg/kg) and placed in the same anesthetic chambers as above with humidified 30% oxygen at 38°C. Mice in the vehicle control group (n = 19) received a peritoneal injection of intralipid (MP Biomedicals, Solon, OH) and placed in the same anesthetic chambers as above with humidified 30% oxygen at room temperature. The concentration of propofol (150 mg/kg) was determined in a pilot study to be sufficient for loss of righting for at least 6 h in P7 mice. Actually, all mice receiving one dose of propofol injection (150 mg/kg, IP) in the formal study maintained loss of righting for 6 hrs and most mice usually recovered the righting or woke up around 8 hr after propofol injection. This dose has also been determined to be the ED_50_ for propofol to maintain an adequate surgical plane of anesthesia, although up to 300 mg/kg, IP has been used for the study of propofol neurotoxicity in infant mouse [46]. Similarly, 1.5% isoflurane is about ED_50_ for P7 mice. The rectal temperature was periodically checked to ensure maintenance of body temperature at 37±0.5°C using a thermometer (Fisher Scientific, Pittsburgh, PA). All mice in the propofol treatment group survived and 2 mice from isoflurane treatment group died. Compared with the control or vehicle mice, the anesthetized mice did not show significant changes in other behaviors after recovery (e.g., eating, drinking and body weight etc.). 11 male and 9 female mice were used for propofol treatment group, while 9 male and 10 female were used for vehicle controls.

A subset of animals from each experimental group was sacrificed 2 h after the anesthetic exposures for blood collection and biochemical assays. The remaining 54 mice were allowed to mature and underwent behavioral testing 32 days post-exposure, which are the required minimum age for mice to perform adequate Morris Water Maze (MWM) tests.

### Measurement of plasma S100β by ELISA

Two hours after the anesthetic exposures, P7 animals were deeply anesthetized with 3% isoflurane for less than 1 min and blood was collected from the left ventricle at the time of sacrifice. S100β levels in the plasma were determined using Sangtec 100 ELISA kits (DiaSorin Inc, Stillwater, MN) following the manufacturer's protocol as we described previously [10], [11]. Briefly, 0.1 ml of blood was centrifuged at 1500 RPM for 10 min to get the plasma. Plasma from each animal (50 µl) was mixed with 150 µl of tracer from the ELISA kit, incubated for 2 h, followed by 3,3′,5,5′ tetramethylbenzidine substrate and stop solution. The optical density was read at 450 nm and the concentrations of the samples were measured using a standard curve.

### Brain tissue harvest

Mice were anesthetized briefly with inhalation of 2–3% isoflurane. The brains were harvested after the blood collection above from the P7 mice, and at the time of sacrifice after the behavioral testing, by intracardiac perfusion through the left ventricle and simultaneous exsanguination through the right atrium with ice-cold phosphate buffered saline (pH 7.4). The left cerebral cortex of each brain was dissected and immediately frozen in liquid nitrogen and stored at −80°C for Western blot analyses. The entire right hemisphere was post-fixed overnight in 4% paraformaldehyde in phosphate buffered saline, embedded in paraffin and serially sectioned (10 µm).

### Western blot

As in our previous studies [10], [13], frozen cortical brain tissue was homogenized on ice using immunoprecipitation buffer (10 mM Tris-HCl, Ph 7.4,150 nM NaCl, 2 mM EDTA, AND 0.5% Nonidet P-40) plus protease inhibitors (1 µg/ml aprotinin, 1 µg/ml leupeptin, and 1 µg/ml pepsstatin A). The lysates were collected, centrifuged at 12,000 rpm for 10 min, and quantified for protein concentration with BCA protein assay kit (Pierce Biotechnology, Rockford, IL). To determine apoptosis in the brain after anesthetic exposures, caspase-3 levels were examined. Briefly, proteins from the P7 mouse cerebral cortex were separated by 12% gel electrophoresis and were transferred to a nitrocellulose membrane. The blots were incubated with a monoclonal antibody against cleaved caspase-3 (1∶1000 dilution; Cell signaling technology, Boston, MA), then probed with horseradish peroxidase-conjugated secondary antibody. To study anesthesia-mediated neuroinflammation, we examined the protein levels of interleukin-1 (IL-1β), interleukin-6 (IL-6) and tumor necrosis factor alpha (TNF-α) by using antibodies to IL-1β (1∶1,000 dilution; Abcam, Cambridge, MA), IL-6 (1∶1,000 dilution; Abcam, Cambridge, MA) and TNF-α (1∶1,000 dilution; Abcam, Cambridge, MA). Detection was performed using the ECL-PLUS system and images were analyzed. Beta actin protein was used as a loading control. The band densities were measured with a GS-800 Densitometer (BIO-RAD, Hercules, CA). and Quantity One software (BIO-RAD version 4.5.0) and averaged for 4 replicates for each animal.

### Immunohistochemistry in brain sections

As we described previously [10], [13], [51], [52], brain sections were deparaffinized, rehydrated with graded alcohols, and washed in distilled water. The sections were incubated with anti-rabbit cleaved caspase-3 primary antibody (1/200, Cell Signaling Technology, Inc Danvers, MA, U.S.A) overnight and the next day incubated with secondary biotinylated goat anti-rabbit antibody (1/200, Santa Cruz Biotechnology, Inc., Santa Cruz, CA) for 40 minutes, followed by incubation with the avidin-biotinylated peroxidase complex (Vectostain ABC-Kit, Vector Lab, Burlingame, CA) for 40 minutes. Negative control sections were incubated in blocking solution without primary antibody. Images were acquired and assessed at 20× using IP lab 7.0 software on an Olympus IX70 microscope (Olympus corporation, Tokyo, Japan) equipped with a Cooke SensiCam camera (Cooke Corp., Romulus, MI). Three brain tissue sections corresponding to the Atlas of the Developing Mouse Brain at P6 Figure 131–133 [53] were chosen from each animal and analyzed for caspase-3 positive cells in the frontal cortex and hippocampal CA1 regions. Two investigators, blinded to the conditions, counted the number of caspase-3 positive cells using IPLab Suite v3.7 imaging processing and analysis software (Biovision Technologies, Exton, PA. The number of caspase-3 positive cells per mm^2^ were quantified for each region. The numbers of animals examined in each group were as follows: isoflurane exposed (n = 6), controls (n = 6), propofol exposed (n = 6) and vehicle controls (n = 6).

### Morris water maze

Learning and memory testing was conducted 32 days after the anesthetic exposures at P34 using the Morris Water Maze (MWM), as we described previously [10], [13], [51]. Briefly, the mice were first trained to escape from the pool (four 60 s trials per day for 5 days). A round plexiglass pool, 150 cm in diameter and 60 cm in height, was filled with water to a height of 1.5 cm above the top of the movable clear 15 cm diameter platform, which was flagged. The pool was covered with a white curtains, water was kept at a temperature between 26°C and 29°C with a pond heater and opacified with titanium dioxide. A video tracking system recorded the time for each animal to reach the platform and the data were analyzed using motion-detection software for the MWM (Actimetrics software, Evanston, IL). After every trial, each mouse was placed in a holding cage, under an infrared heat lamp, before returning to its home cage. The cued trials were used to determine any non-cognitive performance impairments (e.g. visual impairments, swimming difficulties).

For the reference memory trials (place trials), all mice received 4 trials per day for 5 days. The curtain was removed from around the pool to reveal numerous visual cues in the room and the submerged platform was hidden. For each trial, mice were placed in the pool at fixed starting points and allowed to search for the platform for up to 60 s. If a mouse did not find the platform within 60 s, it was gently guided to the platform and allowed to remain there for 30 s. Mice that found the platform also remained on it for 30 s before removal from the pool. The escape latency (time for each mouse to reach the hidden platform) was recorded.Spatial learning ability was reflected by the escape latency; the less time it took to reach the platform, the better spatial learning ability. The mice received two blocks of trials (two trials per block with 30 s apart, 60 s maximum for each trial, with 2 h rest between blocks) each day for 5 days.

Immediately after the place trials and 24 h later, probe trials were conducted to evaluate memory retention. The platform was removed from the pool and the mouse was placed in the opposite quadrant. Each mouse was allowed to swim 60 s and the time spent in each quadrant and the swim speed were recorded and analyzed. The data are expressed as the percent time spent in each of the four quadrants.

### Statistical analysis

ELISAs, western blots and immunohistochemical assays were analyzed using one-way ANOVA followed by post hoc student-Newman-Keuls test. Behavioral studies were analyzed with two-way ANOVA with repeated measures followed by the Bonferroni Multiple Comparison Test using pair-wise comparisons. There were no missing data for the variables during the data analyses. Values of P<0.05 were considered statistically significant. GraphPad Prism 5 software (GraphPad Software, Inc., San Diego, CA) was used for all statistical analysis and graph generation. Animal numbers in each experimental group are listed in the figure legends. Biochemical assay data were expressed as mean±SD and behavioral data were expressed as mean±SEM.

## Results

### Isoflurane but not propofol significantly increased plasma S100β

**Figure 1:**
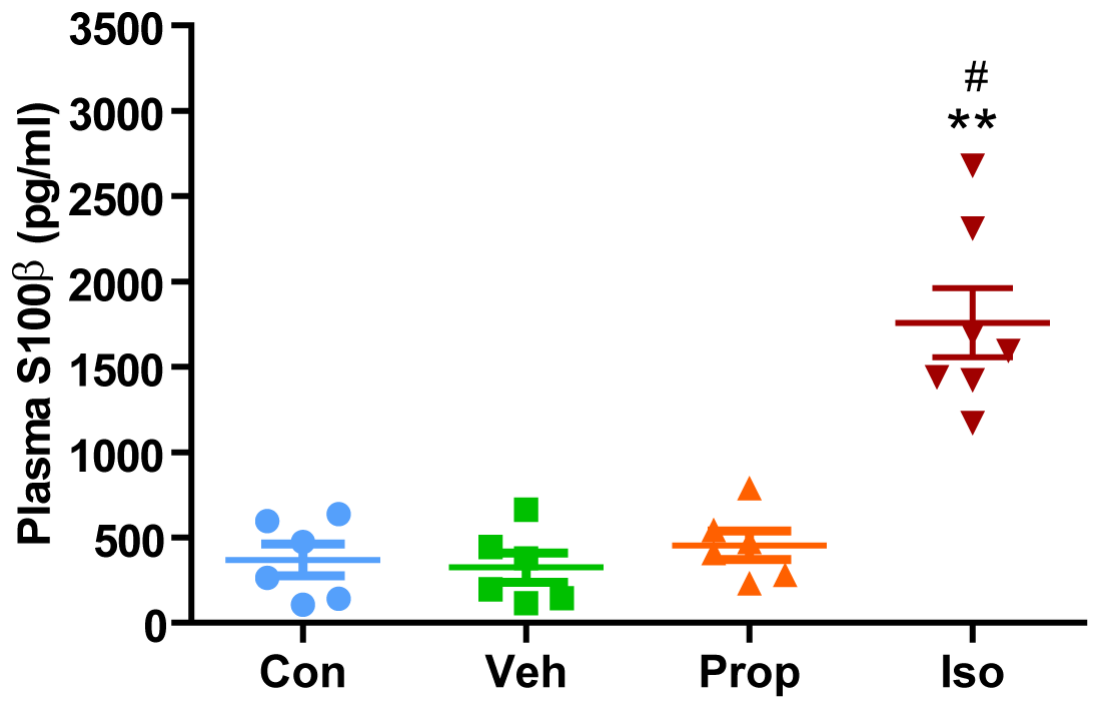
Isoflurane, but not propofol, significantly increases plasma S100β level in P7 mice. Quantitation of ELISAs from the P7 cerebral cortex after 6(n = 6), propofol (n = 6), 30% oxygen (control, n = 6),or intralipid (vehicle, n = 7) revealed that S100β levels were only significantly increased after exposure to isoflurane. Data are expressed as mean±SD and were analyzed by one-way ANOVA followed by the student-Newman-Keuls post hoc test. **P<0.01 compared to control, #P<0.05 compared to propofol. Con (control), Veh (vehicle), Prop (propofol), Iso (isoflurane).

S100β has been shown to be a useful biomarker for the detection of anesthetic-mediated neurodegeneration [10], [11]. As demonstrated in Fig. 1, exposure to 1.5% isoflurane for 6 h in P7 mice significantly increased plasma S100β levels compared to controls, while exposure to propofol for 6 h did not increase plasma S100β levels significantly.

### Isoflurane induced greater apoptosis than propofol in the developing brain

**Figure 2:**
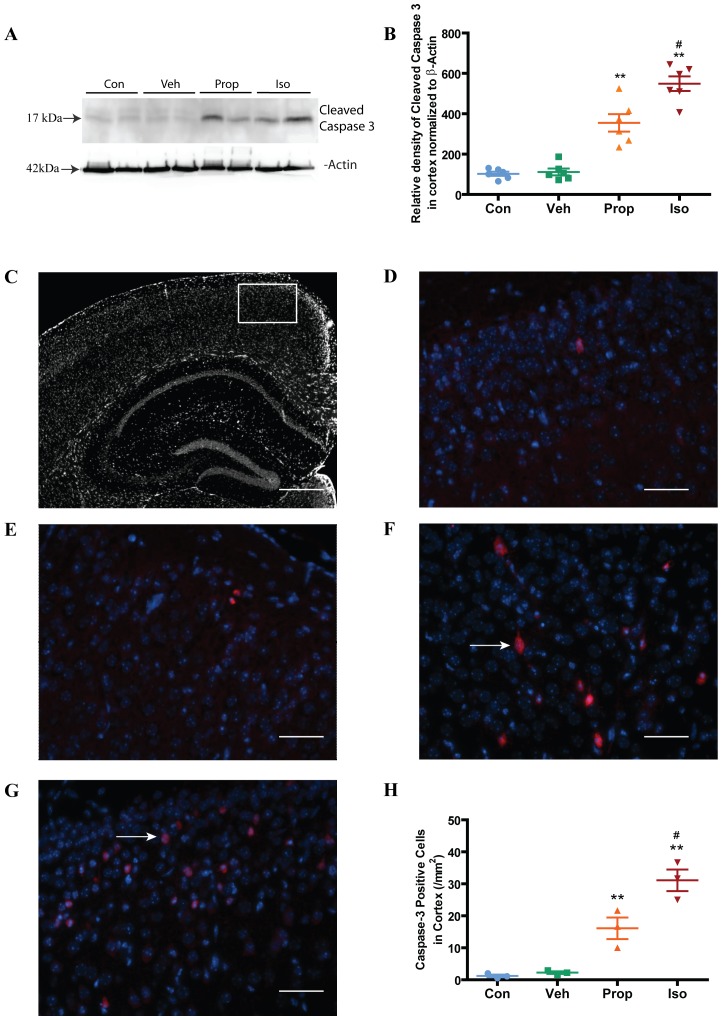
Isoflurane induced significantly greater apoptosis than propofol in cortex of P7 mice. (**A**) Representative western blot from cortex after exposures to isoflurane or propofol in P7 mice. (**B**) Quantification of the Western blots showed that though both isoflurane and propofol exposures significantly increased apoptosis in the cortex, isoflurane was more potent. (**C**) Image of the area sampled in the cortex (white rectangle) for cleaved-caspase-3 positive cells. Scale bar = 600 µm. (**D and E**) Examples of cortical images from control and vehicle groups, respectively. Scale bar = 50 µm. (**F and G**) Examples of caspase-3 positive cells (red stained, white arrows) in the cortex of P7 mice exposed to isoflurane and propofol, respectively. Scale bar = 50 µm. (**H**) Quantitation of the number of caspase-3 positive cells per area from the various experimental groups showed that both isoflurane and propofol significantly increased apoptosis in the cortex and that isoflurane had a significantly greater effect than propofol. All Western blot assay data are expressed as mean±SD (n = 6 in each group, 4 replicates per animal) and were analyzed by one-way ANOVA followed by Newman-Keuls post hoc test. The immunohistochemical data are expressed as number per area and analyzed by one-way ANOVA followed by Newman-Keuls post hoc test, mean+SE (n = 6 in each group, 3 sections per animal). ** P<0.01 compared to control or vehicle respectively, **^#^** P<0.05 compared to the propofol group. Con (control), Veh (vehicle), Prop (propofol), Iso (isoflurane).

**Figure 3:**
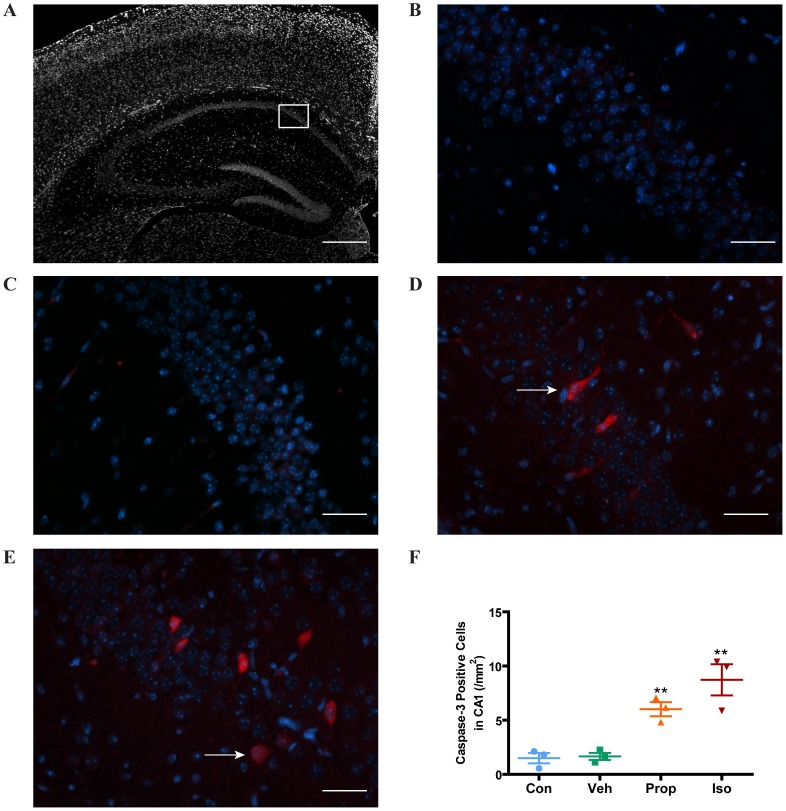
Isoflurane and propofol induced apoptosis in the hippocampal CA1 region of P7 mice. (**A**) Are of the hippocampal CA1 region sampled is demonstrated with the white rectangle. Scale bar = 600 µm. (**B and C**). Representative images of the hippocampal CA1 region from control and vehicle control groups, respectively. Scale bar = 50 µm. (**D and E**). Examples of caspase-3 positive cells (red stained, white arrows) are demonstrated in the hippocampal CA1 region after isoflurane or propofol treatments, respectively. Scale bar = 50 µm. (**F**) Quantitation of the caspase-3 positive cells per mm^2^ from the experimental groups showed that both isoflurane and propofol significantly increased apoptosis in the hippocampus compared to the control groups. Data are expressed as mean+SE (n = 6 in each group, 3 sections per animal) and were analyzed by one-way ANOVA followed by Newman-Keuls post hoc test. ** P<0.01 compared to control or vehicle respectively.

Caspase-3 is cleaved during the process of apoptosis and the cleaved caspase-3 is a well accepted biomarker for cell death by apoptosis. We investigated the effects of exposure to isoflurane or propofol on cleaved caspase-3 in the cortex and hippocampus in 7 day-old mice. We found that while both isoflurane and propofol significantly increased cleaved caspase-3 levels in the cortex determined by Western blot (Fig. 2 A and B) and immunohistochemistry (Fig. 2 C–G), isoflurane was more potent than propofol. Similarly, immunohistochemical analysis of the hippocampal CA1 region (Fig. 3) showed that both isoflurane and propofol significantly increased cleaved caspase-3.

### Effects of isoflurane and propofol on inflammation

**Figure 4:**
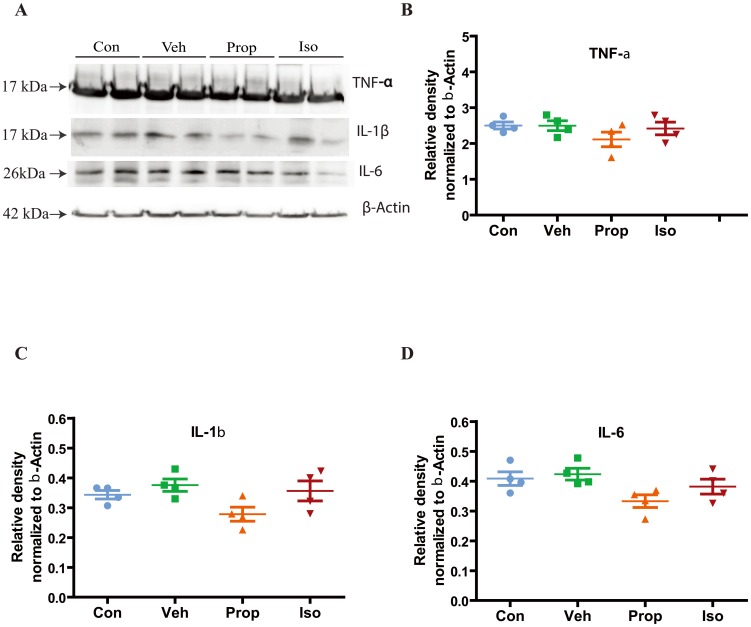
Effects of isoflurane and propofol on inflammation. (**A**) A representive Western blot of TNF-α, IL-6 and IL-1β levels in the P7 cortex immediately after anesthetic exposures. IL = interleukin; TNF = tumor necrosis factor. (**B–D**) Quantification of the western blot data normalized to β-actin for TNF-α, IL-6 and IL-1β levels respectively in the mouse cortex at P7. Data are expressed as mean+SD (n = 6 in each group, 4 replicate blots per animal) and were analyzed by one-way ANOVA followed by Newman-Keuls post hoc test.

Given that recent studies have suggested that isoflurane may induce neuroinflammation which may be associated with cognitive impairment [12], [35], [48], we examined with Western blot assays (Fig. 4A) the effects of isoflurane and propofol on the proinflammatory cytokines, TNF-α (Fig. 4B), IL-1β (Fig. 4C) and IL-6 (Fig. 4D), in the cortex at P7. Neither isoflurane nor propofol significantly changed these markers of inflammation.

### Effects of isoflurane and propofol on cognitive function

**Figure 5:**
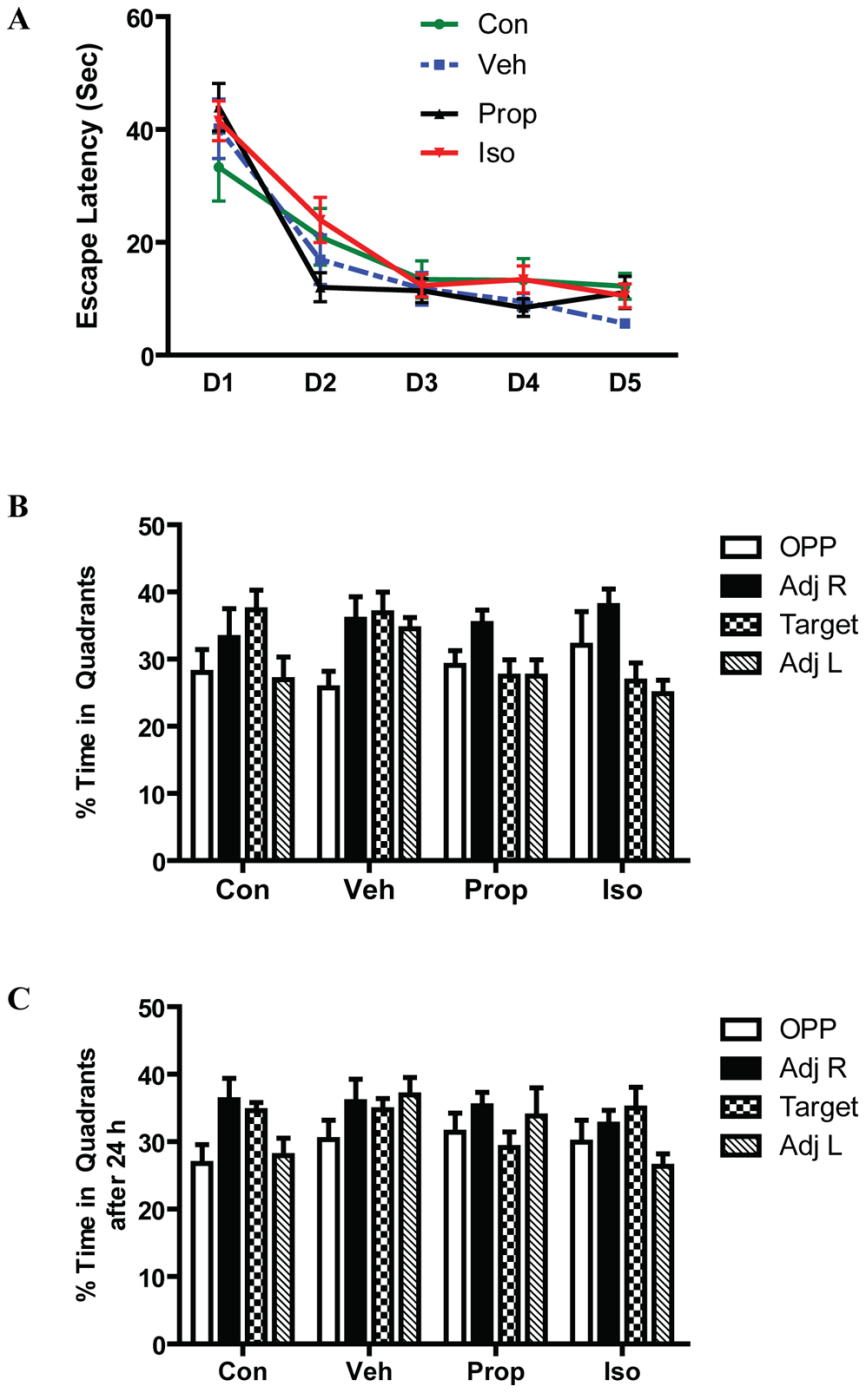
Effects of isoflurane and propofol on cognitive function. (**A**) The mean escape latency over 5 consecutive days (D) of place trials showed no significant difference in all groups. Probe trials were conducted to measure memory retention. The percent time spent in each quadrant was determined immediately (**B**) or 24 hr (**C**) after the last place trial. Data were analyzed by two-way ANOVA with repeated measures. All data represent mean ± SEM. Con, Control (n = 12), Veh, vehicle (n = 12), Prop, propofol (n = 14), Iso, isoflurane (n = 16).

The Morris Water Maze test was used to evaluate potential learning and memory deficits after exposure to isoflurane and propofol in mice 32 days after anesthesia exposure at P7. The cued and reference memory trials demonstrated no significant physical impairments in four groups (data not shown). The escape latency in place trials, shown in Fig. 5A, indicated that the average time to reach the submerged platform did not differ significantly after exposure to isoflurane or propofol. Probe trials demonstrated no statistical difference in retention memory either immediately (Fig. 5B) after the place trials or 24 h later (Fig. 5C). In addition, there were no significant differences in swim speed during the probe trials (data not shown).

## Discussion

This study showed that even though isoflurane and propofol significantly increased neuroapoptosis in the rodent developing brain, this was not associated with immediate changes in neuorinflammation or subsequent cognitive dysfunction.

Commonly used general anesthetics have been reported to cause apoptosis in the developing brain and subsequent cognitive dysfunction [1], [2], [48], [49]. One strategy to minimize these possible detrimental effects of GAs is to use a less neurotoxic GA in the more vulnerable developing brain. Many studies have compared the potency of isoflurane, sevoflurane and desflurane to cause neurodegeneration in the brain, with controversial results [10], [12], [48], [49], [54], [55]. Some studies demonstrated a higher potency of isoflurane to cause neuroapoptosis compared to sevoflurane [10] or desflurane [48], while others could not confirm these findings [49], [54]. The variation may be caused by different ways to determine the minimal alveolar concentration (MAC), either with [49], [54] or without [10], [12], [48] a stimulus to pinch the tail during anesthesia exposure, as well as different animal species and experimental protocols [10], [12], [48], [49], [54], [55]. Compared to inhalational anesthetics, fewer studies have examined the neurotoxicity of the commonly used intravenous anesthetic, propofol. Recent studies suggest that propofol, like inhalational anesthetics, also induced apoptosis of neurons and oligodendrocytes during brain development in rhesus macaques [4], rats [7], [47] and mice [46], and are associated with cognitive dysfunction, especially after repeated exposures [7]. The mechanism of propofol-mediated apoptosis in the developing brain is still unclear, but it is likely to share common mechanisms with inhalational general anesthetics, such as GABA receptor activation [25], P75 neurotrophin receptor activation [5], [30], and intracellular calcium dysregulation by over activation of inositol trisphosphate (InsP_3_) receptors [13], [15], [56]. Few studies have directly compared the potency of neurotoxicity of propofol and an inhalational anesthetic in the developing brain, although exposure to sevoflurane has poorer cognitive outcomes than propofol in elderly patients with mild cognitive dysfunction [57]. We have found propofol to be less effective than isoflurane in activating InsP_3_ receptors and therefore propofol may induce less over-activation of the InsP_3_ receptor and less apoptosis accordingly [13], [15], [19]. Results from this study suggest that propofol is less potent than isoflurane in causing neurodegeneration in the developing brain, evidenced by no changes in plasma S100β levels and less significant caspase-3 activation in the cortex. The possible clinical implications of these results need to be further studied.

Recent studies suggest that inflammation may play an important role in GA-mediated neurodegeneration, especially its association with cognitive dysfunction [12], [35], [48]. Interestingly, GAs also have different potencies in affecting neuroinflammation and, thus, cognitive dysfunction [12], [48]. However, the present study demonstrates significant apoptosis induced by isoflurane and propofol without significant changes in inflammation, suggesting other mechanisms may play important roles in GA-induce apoptosis in this animal model. Although initial studies suggested an association between isoflurane-mediated apoptosis in the developing brain and subsequent cognitive dysfunction [1], [2], further studies could not confirm this association, at least at low concentrations of isoflurane [10], [13], [58]. On the other hand, GA-mediated inflammation has been demonstrated to correlate well with cognitive dysfunction [12], [48]. This hypothesis is supported by the close association between surgery induced inflammation and postoperative cognitive dysfunction [38]–[41]. Results from this study also support these findings, in that while both propofol and isoflurane caused significant apoptosis in the developing brain, this did not correlate with neuroinflammation or later cognitive dysfunction. Further studies including surgery are needed to confirm that inflammation during brain development may play a more important role in GA-mediated cognitive dysfunction than apoptosis. It has been shown that propofol may exert its neuroprotective effects in animal models of ischemia [59], trauma [60] and pressure stress [61] by limiting microglia activation. In contrast, propofol treatment alone without other stresses factors did not induce inflammation [62], and demonstrated significant induction of apoptosis in the infant mouse brains in the current study. The role of microglia activation in propofol induced apoptosis in the developing brains need to be studied furthermore in the future.

It has been widely accepted that general anesthetics cause significant apoptosis in the developing brain in various species. Our previous studies [10], [11] have demonstrated a close relationship between isoflurane induced apoptosis in the rodent developing brain and the elevation of plasma S100β, a sensitive but not specific neurodegenerative biomarker, to detect brain damage induced by various stresses [63], [64]. Similar to our previous studies [10], [11], the plasma S100β measured in this study most likely is reflective of the exposure to isoflurane because no surgeries or other significant stresses had been applied to these animals. However, while propofol significantly induced apoptosis in the developing brain, it failed to increase plasma S100β levels. This could be due to S100β not being as sensitive of a marker of neurodegeneration in the developing brain as caspase-3. Further studies in both animals and human beings are needed to investigate the value of plasma S100β as a biomarker to detect anesthetic-mediated neurodegeneration in the developing brain.

Our study has several limitations. We did not measure the actual plasma concentrations of isoflurane and propofol as it is technically difficult to place an intravenous line and obtain blood in P7 mice. Likewise, we did not carry out continuous intravenous infusions of propofol to maintain a constant concentration of propofol in the blood. Instead, we used the ED_50_ dose to maintain a surgical plane of anesthesia and shown to cause significant apoptosis in the mouse developing brain [46]. We only exposed the mice to anesthesia, without surgical stimulation, which does not truly mimic the clinical scenario. Recent studies have suggested that surgery, rather than anesthesia, plays an important role in postoperative cognitive dysfunction, possibly by promoting neuroinflammation [40], [52]. To minimize the use of animals, we did not do a dose-response study for the isoflurane and propofol treatments, which may provide more insight into their effects on apoptosis, inflammation and cognitive function.

In summary, we have demonstrated that a onetime 6 h clinically relevant exposure to isoflurane or propofol induced significant apoptosis in the mouse P7 developing brain, with isoflurane being more potent, which was not associated with significant changes in neuroinflammation or cognitive function.

## References

[1] Jevtovic-Todorovic V, Hartman RE, Izumi Y, Benshoff ND, Dikranian K, et al (2003) Early exposure to common anesthetic agents causes widespread neurodegeneration in the developing rat brain and persistent learning deficits. J Neurosci 23: 876–882. 12574416 10.1523/JNEUROSCI.23-03-00876.2003 PMC6741934

[2] Ma D, Williamson P, Januszewski A, Nogaro MC, Hossain M, et al (2007) Xenon mitigates isoflurane-induced neuronal apoptosis in the developing rodent brain. Anesthesiology 106: 746–753. 17413912 10.1097/01.anes.0000264762.48920.80

[3] Slikker W, Zou XJ, Hotchkiss CE, Divine RL, Sadovova N, et al (2007) Ketamine-induced neuronal cell death in the perinatal rhesus monkey. Toxicol Sci 98: 145–158. 17426105 10.1093/toxsci/kfm084

[4] Creeley C, Dikranian K, Dissen G, Martin L, Olney J, et al (2013) Propofol-induced apoptosis of neurones and oligodendrocytes in fetal and neonatal rhesus macaque brain. Br J Anaesth 110 Suppl 1: i29–i38 10.1093/bja/aet173 23722059 PMC3667347

[5] Pearn ML, Hu Y, Niesman IR, Patel HH, Drummond JC, et al (2012) Propofol neurotoxicity is mediated by p75 neurotrophin receptor activation. Anesthesiology 116: 352–361 10.1097/ALN.0b013e318242a48c 22198221 PMC3275822

[6] Tu S, Wang X, Yang F, Chen B, Wu S, et al (2011) Propofol induces neuronal apoptosis in infant rat brain under hypoxic conditions. Brain Res Bull 86: 29–35 10.1016/j.brainresbull.2011.06.017 21756983

[7] Yu D, Jiang Y, Gao J, Liu B, Chen P (2013) Repeated exposure to propofol potentiates neuroapoptosis and long-term behavioral deficits in neonatal rats. Neurosci Lett 534: 41–46 10.1016/j.neulet.2012.12.033 23295901

[8] Brambrink AM, Evers AS, Avidan MS, Farber NB, Smith DJ, et al (2010) Isoflurane-induced neuroapoptosis in the neonatal rhesus macaque brain. Anesthesiology 112: 834–841 10.1097/ALN.0b013e3181d049cd 20234312 PMC3962067

[9] Zou X, Liu F, Zhang X, Patterson TA, Callicott R, et al (2011) Inhalation anesthetic-induced neuronal damage in the developing rhesus monkey. Neurotoxicol Teratol 33: 592–597 10.1016/j.ntt.2011.06.003 21708249

[10] Liang G, Ward C, Peng J, Zhao Y, Huang B, et al (2010) Isoflurane causes greater neurodegeneration than an equivalent exposure of sevoflurane in the developing brain of neonatal mice. Anesthesiology 112: 1325–1334 10.1097/ALN.0b013e3181d94da5 20460994 PMC2877765

[11] Wang S, Peretich K, Zhao Y, Liang G, Meng Q, et al (2009) Anesthesia-induced neurodegeneration in fetal rat brains. Pediatr Res 66: 435–440. 20016413 10.1203/PDR.0b013e3181b3381b PMC3069715

[12] Shen X, Dong Y, Xu Z, Wang H, Miao C, et al (2013) Selective anesthesia-induced neuroinflammation in developing mouse brain and cognitive impairment. Anesthesiology 118: 502–515 10.1097/ALN.0b013e3182834d77 23314110 PMC3580002

[13] Zhao Y, Liang G, Chen Q, Joseph DJ, Meng Q, et al (2010) Anesthetic-induced neurodegeneration mediated via inositol 1,4,5-trisphosphate receptors. J Pharmacol Exp Ther 333: 14–22 10.1124/jpet.109.161562 20086058 PMC2846020

[14] Flick RP, Katusic SK, Colligan RC, Wilder RT, Voigt RG, et al (2011) Cognitive and behavioral outcomes after early exposure to anesthesia and surgery. Pediatrics 128: e1053–e1061 10.1542/peds.2011-0351 21969289 PMC3307194

[15] Wei HF, Liang G, Yang H, Wang QJ, Hawkins B, et al (2008) The common inhalational anesthetic isoflurane induces apoptosis via activation of inositol 1,4,5-trisphosphate receptors. Anesthesiology 108: 251–260. 18212570 10.1097/01.anes.0000299435.59242.0e

[16] Lunardi N, Ori C, Erisir A, Jevtovic-Todorovic V (2010) General anesthesia causes long-lasting disturbances in the ultrastructural properties of developing synapses in young rats. Neurotox Res 17: 179–188 10.1007/s12640-009-9088-z 19626389 PMC3629551

[17] Sanchez V, Feinstein SD, Lunardi N, Joksovic PM, Boscolo A, et al (2011) General Anesthesia Causes Long-term Impairment of Mitochondrial Morphogenesis and Synaptic Transmission in Developing Rat Brain. Anesthesiology 115: 992–1002 10.1097/ALN.0b013e3182303a63 21909020 PMC3203321

[18] Liang G, Wang QJ, Li Y, Kang B, Eckenhoff MF, et al (2008) A presenilin-1 mutation renders neurons vulnerable to isoflurane toxicity. Anesth Analg 106: 492–500. 18227305 10.1213/ane.0b013e3181605b71

[19] Yang H, Liang G, Hawkins BJ, Madesh M, Pierwola A, et al (2008) Inhalational anesthetics induce cell damage by disruption of intracellular calcium homeostasis with different potencies. Anesthesiology 109: 243–250. 18648233 10.1097/ALN.0b013e31817f5c47 PMC2598762

[20] Wei H, Xie Z (2009) Anesthesia, calcium homeostasis and Alzheimer's disease. Curr Alzheimer Res 6: 30–35. 19199872 10.2174/156720509787313934 PMC2735723

[21] Wang Q, Liang G, Yang H, Wang S, Eckenhoff MF, et al (2011) The common inhaled anesthetic isoflurane increases aggregation of huntingtin and alters calcium homeostasis in a cell model of Huntington's disease. Toxicol Appl Pharmacol 250: 291–298 10.1016/j.taap.2010.10.032 21059370 PMC3022103

[22] Wei H (2011) The role of calcium dysregulation in anesthetic-mediated neurotoxicity. Anesth Analg 113: 972–974 10.1213/ANE.0b013e3182323261 22021793 PMC3201768

[23] Zhao X, Yang Z, Liang G, Wu Z, Peng Y, et al (2013) Dual Effects of Isoflurane on Proliferation, Differentiation, and Survival in Human Neuroprogenitor Cells. Anesthesiology 118: 537–549 10.1097/ALN.0b013e3182833fae 23314167 PMC3580019

[24] Zhang GH, Dong YL, Zhang B, Ichinose F, Wu X, et al (2008) Isoflurane-induced caspase-3 activation is dependent on cytosolic calcium and can be attenuated by memantine. J Neurosci 28: 4551–4560. 18434534 10.1523/JNEUROSCI.5694-07.2008 PMC3844798

[25] Zhao YL, Xiang Q, Shi QY, Li SY, Tan L, et al (2011) GABAergic excitotoxicity injury of the immature hippocampal pyramidal neurons' exposure to isoflurane. Anesth Analg 113: 1152–1160 10.1213/ANE.0b013e318230b3fd 21918167

[26] Sinner B, Friedrich O, Zink W, Zausig Y, Graf BM (2011) The toxic effects of s(+)-ketamine on differentiating neurons in vitro as a consequence of suppressed neuronal Ca2+ oscillations. Anesth Analg 113: 1161–1169 10.1213/ANE.0b013e31822747df 21788311

[27] Istaphanous GK, Ward CG, Nan X, Hughes EA, Mccann JC, et al (2013) Characterization and quantification of isoflurane-induced developmental apoptotic cell death in mouse cerebral cortex. Anesth Analg 116: 845–854 10.1213/ANE.0b013e318281e988 23460572

[28] Ikonomidou C, Bittigau P, Koch C, Genz K, Hoerster F, et al (2001) Neurotransmitters and apoptosis in the developing brain. Biochem Pharmacol 62: 401–405. 11448448 10.1016/s0006-2952(01)00696-7

[29] Brambrink AM, Evers AS, Avidan MS, Farber NB, Smith DJ, et al (2012) Ketamine-induced neuroapoptosis in the fetal and neonatal rhesus macaque brain. Anesthesiology 116: 372–384 10.1097/ALN.0b013e318242b2cd 22222480 PMC3433282

[30] Head BP, Patel HH, Niesman IR, Drummond JC, Roth DM, et al (2009) Inhibition of p75 neurotrophin receptor attenuates isoflurane-mediated neuronal apoptosis in the neonatal central nervous system. Anesthesiology 110: 813–825. 19293698 10.1097/ALN.0b013e31819b602b PMC2767332

[31] Soriano SG, Liu Q, Li J, Liu JR, Han XH, et al (2010) Ketamine activates cell cycle signaling and apoptosis in the neonatal rat brain. Anesthesiology 112: 1155–1163 10.1097/ALN.0b013e3181d3e0c2 20418696

[32] Zou X, Sadovova N, Patterson TA, Divine RL, Hotchkiss CE, et al (2008) The effects of L-carnitine on the combination of, inhalation anesthetic-induced developmental, neuronal apoptosis in the rat frontal cortex. Neuroscience 151: 1053–1065. 18201836 10.1016/j.neuroscience.2007.12.013

[33] Yon JH, Carter LB, Reiter RJ, Jevtovic-Todorovic V (2006) Melatonin reduces the severity of anesthesia-induced apoptotic neurodegeneration in the developing rat brain. Neurobiol Dis 21: 522–530. 16289675 10.1016/j.nbd.2005.08.011

[34] Cao L, Li L, Lin D, Zuo Z (2012) Isoflurane induces learning impairment that is mediated by interleukin 1beta in rodents. PLoS One 7: e51431 10.1371/journal.pone.0051431 23251531 PMC3520904

[35] Wu X, Lu Y, Dong Y, Zhang G, Zhang Y, et al (2012) The inhalation anesthetic isoflurane increases levels of proinflammatory TNF-alpha, IL-6, and IL-1beta. Neurobiol Aging 33: 1364–1378 10.1016/j.neurobiolaging.2010.11.002 21190757 PMC3117127

[36] Tang JX, Eckenhoff MF, Eckenhoff RG (2011) Anesthetic modulation of neuroinflammation in Alzheimer's disease. Curr Opin Anaesthesiol 24: 389–394 10.1097/ACO.0b013e32834871c5 21659873 PMC3289136

[37] Lin D, Zuo Z (2011) Isoflurane induces hippocampal cell injury and cognitive impairments in adult rats. Neuropharmacology 61: 1354–1359 10.1016/j.neuropharm.2011.08.011 21864548 PMC3189329

[38] Tang JX, Mardini F, Janik LS, Garrity ST, Li RQ, et al (2012) Modulation of Murine Alzheimer Pathogenesis and Behavior by Surgery. Ann Surg 10.1097/SLA.0b013e318269d623 PMC3525732 22964728

[39] Terrando N, Monaco C, Ma D, Foxwell BM, Feldmann M, et al (2010) Tumor necrosis factor-alpha triggers a cytokine cascade yielding postoperative cognitive decline. Proc Natl Acad Sci U S A 107: 20518–20522 10.1073/pnas.1014557107 21041647 PMC2996666

[40] Terrando N, Eriksson LI, Ryu JK, Yang T, Monaco C, et al (2011) Resolving postoperative neuroinflammation and cognitive decline. Ann Neurol 70: 986–995 10.1002/ana.22664 22190370 PMC4556354

[41] Feng X, Degos V, Koch LG, Britton SL, Zhu Y, et al (2013) Surgery results in exaggerated and persistent cognitive decline in a rat model of the Metabolic Syndrome. Anesthesiology 118: 1098–1105 10.1097/ALN.0b013e318286d0c9 23353794 PMC5530762

[42] Loepke AW (2010) Developmental neurotoxicity of sedatives and anesthetics: a concern for neonatal and pediatric critical care medicine? Pediatr Crit Care Med 11: 217–226 10.1097/PCC.0b013e3181b80383 19770789

[43] Sun L (2010) Early childhood general anaesthesia exposure and neurocognitive development. Br J Anaesth 105 Suppl 1: i61–i68 10.1093/bja/aeq302 21148656 PMC3000523

[44] Sanders RD, Sun P, Patel S, Li M, Maze M, et al (2010) Dexmedetomidine provides cortical neuroprotection: impact on anaesthetic-induced neuroapoptosis in the rat developing brain. Acta Anaesthesiol Scand 54: 710–716 10.1111/j.1399-6576.2009.02177.x 20003127

[45] Brambrink AM, Back SA, Riddle A, Gong X, Moravec MD, et al (2012) Isoflurane-induced apoptosis of oligodendrocytes in the neonatal primate brain. Ann Neurol 72: 525–535 10.1002/ana.23652 23109147 PMC3490441

[46] Cattano D, Young C, Straiko MM, Olney JW (2008) Subanesthetic doses of propofol induce neuroapoptosis in the infant mouse brain. Anesth Analg 106: 1712–1714 10.1213/ane.0b013e318172ba0a 18499599

[47] Tu S, Wang X, Yang F, Chen B, Wu S, et al (2011) Propofol induces neuronal apoptosis in infant rat brain under hypoxic conditions. Brain Res Bull 86: 29–35 10.1016/j.brainresbull.2011.06.017 21756983

[48] Zhang Y, Xu Z, Wang H, Dong Y, Shi HN, et al (2012) Anesthetics isoflurane and desflurane differently affect mitochondrial function, learning, and memory. Ann Neurol 71: 687–698 10.1002/ana.23536 22368036 PMC3942786

[49] Kodama M, Satoh Y, Otsubo Y, Araki Y, Yonamine R, et al (2011) Neonatal desflurane exposure induces more robust neuroapoptosis than do isoflurane and sevoflurane and impairs working memory. Anesthesiology 115: 979–991 10.1097/ALN.0b013e318234228b 21956042

[50] Xie Z, Culley DJ, Dong Y, Zhang G, Zhang B, et al (2008) The common inhalation anesthetic isoflurane induces caspase activation and increases amyloid beta-protein level in vivo. Ann Neurol 64: 618–627 10.1002/ana.21548 19006075 PMC2612087

[51] Li Y, Liang G, Wang S, Meng Q, Wang Q, et al (2007) Effect of fetal exposure to isoflurane on postnatal memory and learning in rats. Neuropharmacology 53: 942–950. 17959201 10.1016/j.neuropharm.2007.09.005 PMC2170454

[52] Tang JX, Mardini F, Janik LS, Garrity ST, Li RQ, et al (2013) Modulation of murine Alzheimer pathogenesis and behavior by surgery. Ann Surg 257: 439–448 10.1097/SLA.0b013e318269d623 22964728 PMC3525732

[53] Paxinos G, Halliday G, Watson C, Koutcherov Y, Wang H (2007) Atlas of the Developing Mouse Brain. Academic Press. 133 p.

[54] Istaphanous GK, Howard J, Nan X, Hughes EA, Mccann JC, et al (2011) Comparison of the neuroapoptotic properties of equipotent anesthetic concentrations of desflurane, isoflurane, or sevoflurane in neonatal mice. Anesthesiology 114: 578–587 10.1097/ALN.0b013e3182084a70 21293251

[55] Valentim AM, Di GP, Ribeiro PO, Rodrigues P, Olsson IA, et al (2010) Lower isoflurane concentration affects spatial learning and neurodegeneration in adult mice compared with higher concentrations. Anesthesiology 113: 1099–1108 10.1097/ALN.0b013e3181f79c7c 20885290

[56] Peng Y, Fosket K, Vais H, Joseph D, Liang G, et al (2011) The general anesthetics facilitate activation of inositol 1,4,5-trisphosphate (InsP3) receptors. Neuroscience Meeting Planner, Society for Neuroscience, Washington, DC Online

[57] Liu Y, Pan N, Ma Y, Zhang S, Guo W, et al (2013) Inhaled sevoflurane may promote progression of amnestic mild cognitive impairment: a prospective, randomized parallel-group study. Am J Med Sci 345: 355–360 10.1097/MAJ.0b013e31825a674d 23044653

[58] Stratmann G, May LDV, Sall JW, Alvi RS, Bell JS, et al (2009) Effect of hypercarbia and isoflurane on brain cell death and neurocognitive dysfunction in 7-day-old rats. Anesthesiology 110: 849–861. 19293696 10.1097/ALN.0b013e31819c7140

[59] Zhou R, Yang Z, Tang X, Tan Y, Wu X, et al (2013) Propofol protects against focal cerebral ischemia via inhibition of microglia-mediated proinflammatory cytokines in a rat model of experimental stroke. PLoS One 8: e82729 10.1371/journal.pone.0082729 24349350 PMC3857282

[60] Luo T, Wu J, Kabadi SV, Sabirzhanov B, Guanciale K, et al (2013) Propofol limits microglial activation after experimental brain trauma through inhibition of nicotinamide adenine dinucleotide phosphate oxidase. Anesthesiology 119: 1370–1388 10.1097/ALN.0000000000000020 24121215

[61] Yu G, Dymond M, Yuan L, Chaturvedi LS, Shiratsuchi H, et al (2011) Propofol's effects on phagocytosis, proliferation, nitrate production, and cytokine secretion in pressure-stimulated microglial cells. Surgery 150: 887–896 10.1016/j.surg.2011.04.002 21676422 PMC3837575

[62] Zhang J, Jiang W, Zuo Z (2014) Pyrrolidine dithiocarbamate attenuates surgery-induced neuroinflammation and cognitive dysfunction possibly via inhibition of nuclear factor kappaB. Neuroscience 261: 1–10 10.1016/j.neuroscience.2013.12.034 24365462 PMC3950371

[63] Bloomfield SM, McKinney J, Smith L, Brisman J (2007) Reliability of S100B in predicting severity of central nervous system injury. Neurocritical Care 6: 121–138. 17522796 10.1007/s12028-007-0008-x

[64] Cata JP, Abdelmalak B, Farag E (2011) Neurological biomarkers in the perioperative period. Br J Anaesth 107: 844–858 10.1093/bja/aer338 22065690

